# Correlation between C-reactive protein and postoperative mortality in patients undergoing hip fracture surgery: a meta-analysis

**DOI:** 10.1186/s13018-023-03516-y

**Published:** 2023-03-09

**Authors:** Bing-Kuan Chen, Yu-Cheng Liu, Chun-Ching Chen, Yu-Pin Chen, Yi-Jie Kuo, Shu-Wei Huang

**Affiliations:** 1 Department of General Medicine, Shuang Ho Hospital, New Taipei City, Taiwan; 2 College of Medicine, Taipei Medical University, Taipei, Taiwan; 3 Department of Orthopedics, Wan Fang Hospital, Taipei Medical University, No. 111, Sec. 3, Xinglong Rd., Wenshan Dist., Taipei City, 116 Taiwan; 4 Department of Orthopaedics, School of Medicine, College of Medicine, Taipei Medical University, Taipei, Taiwan

**Keywords:** Hip fracture, Hip fracture surgery, C-reactive protein, Mortality

## Abstract

**Background:**

Hip fracture is a common but devastating disease with a high mortality rate in the older adult population. C-reactive protein (CRP) is a predictor of the prognosis in many diseases, but its correlations with patient outcomes following hip fracture surgery remain unclear. In this meta-analysis, we investigated the correlation between perioperative CRP level and postoperative mortality in patients undergoing hip fracture surgery.

**Methods:**

PubMed, Embase, and Scopus were searched for relevant studies published before September 2022. Observational studies investigating the correlation between perioperative CRP level and postoperative mortality in patients with hip fracture were included. The differences in CRP levels between the survivors and nonsurvivors following hip fracture surgery were measured with mean differences (MDs) and 95% confidence intervals (CIs).

**Results:**

Fourteen prospective and retrospective cohort studies comprising 3986 patients with hip fracture were included in the meta-analysis. Both the preoperative and postoperative CRP levels were significantly higher in the death group than in the survival group when the follow-up duration was ≥ 6 months (MD: 0.67, 95% CI: 0.37–0.98, *P* < 0.0001; MD: 1.26, 95% CI: 0.87–1.65, *P* < 0.00001, respectively). Preoperative CRP levels were significantly higher in the death group than in the survival group when the follow-up duration was ≤ 30 days (MD: 1.49, 95% CI: 0.29–2.68; *P* = 0.01).

**Conclusions:**

Both higher preoperative and postoperative CRP levels were correlated with higher risk of mortality following hip fracture surgery, suggesting the prognostic role of CRP. Further studies are warranted to confirm the ability of CRP to predict postoperative mortality in patients with hip fracture.

**Supplementary Information:**

The online version contains supplementary material available at 10.1186/s13018-023-03516-y.

## Background

Hip fracture is a common but devastating disease in the older adult population. Although the incidence varies in different places, the correlation between advanced age and higher incidence has been observed in every race [1]. With increases in life expectancy and the size of the older adult population, studies have projected an increasing annual incidence of hip fracture from 1.26–1.66 million in 1990 to 4.50–6.26 million in 2050 [2, 3]. Therefore, hip fracture, which incurs considerable costs due to long periods of hospitalization and rehabilitation, imposes a major burden on public health systems [4].

Hip fractures can be categorized into extracapsular and intracapsular fractures, and different intervention strategies would be adopted based on the fracture types [5]. Displaced and unstable femoral neck fractures account for the majority of intracapsular fractures; surgical interventions such as hemiarthroplasty are often required [6]. For extracapsular fractures, implants including intramedullary nail and dynamic hip screw are chosen and applied depending on the integrity of lateral femur wall [5]. Despite the advances in the knowledge of surgical techniques and patient care in recent decades, the mortality rate in the first year following hip fracture surgery still remains high (13–36%), which indicates hip fracture as a threat to the vulnerable older adult population [7–10]. A reliable method for predicting prognosis is essential for distributing limited medical resources and exploring the pathophysiology underlying this considerable mortality.

Studies have identified numerous factors for predicting mortality following hip fracture surgery, including advanced age, male sex, and multiple comorbidities [9, 11, 12]. Inflammation is also a potential predictor of postoperative mortality in patients undergoing hip fracture surgery. Several biomarkers and indices based on such biomarkers that are used to assess inflammation—including interleukin-6 level, the neutrophil-to-lymphocyte ratio, and the systemic immune-inflammation index—have been reported to be correlated with postoperative mortality in patients with hip fracture [13–15].

C-reactive protein (CRP) is a widely used, easily gathered, and sensitive biomarker for detecting inflammation in clinical practice. Some studies have reported CRP to be an independent prognostic predictor for COVID-19 and numerous types of cancer [16, 17]. Regarding the prognostic role of CRP in hip fracture, numerous studies have reported a correlation between higher perioperative CRP level and higher mortality rate in patients following hip fracture surgery [18–20]; however, some studies did not report such a correlation [21, 22]. No consensus has been reached on the correlation between CRP and hip fracture mortality.

Considering the limited and inconclusive state of the literature, we performed a meta-analysis to explore the correlation of CRP with postoperative mortality in patients undergoing hip fracture surgery. We hypothesized that a higher perioperative CRP level was correlated with higher short- and long-term mortality rates following hip fracture surgery.

## Methods

### Study design and identification of eligible studies

This study was performed in compliance with the Preferred Reporting Items for Systematic Reviews and Meta-analyses statement [23]. Two main reviewers (Y.-C.L. and B.-K.C.) comprehensively searched the PubMed, Embase, and Scopus databases for relevant studies published before September 2022. The following search keywords were employed: (“c-reactive protein” or “CRP”) and (“hip fracture” or “hip fractures” or “hip fracture mortality”). No language restrictions were applied. To broaden the search, the reference lists of relevant studies were screened, and the “related articles” option in PubMed was also used. This meta-analysis was registered in the International Prospective Register of Systematic Reviews (registration no. CRD42022364365).

### Inclusion and exclusion criteria

The inclusion criteria were as follows: (1) observational studies with full texts available, (2) observational studies evaluating the prognostic value of CRP for short- or long-term postoperative mortality following hip fracture surgery, and (3) studies providing complete CRP data. Hip fractures have four subtypes classified in accordance with fracture location, namely femoral head, femoral neck, intertrochanteric, and subtrochanteric fractures.

The exclusion criteria were as follows: (1) studies including patients with multiple traumas, fractures, or pathological fracture, (2) studies including patients who were suspected to have or received a diagnosis of bacterial infection, and (3) studies providing incomplete or invalid information on CRP and the study endpoint.

### Data extraction and appraisal of methodological quality

Two main reviewers (Y.-C.L. and B.-K.C.) independently identified potentially relevant studies, reviewed the full texts of the articles, and extracted baseline and outcome data from either the datasets or figures and tables. The extracted data included the study timeframe, publication year, country, study design, models for predicting postoperative hip fracture mortality, sample size, mean age, sex distribution, primary endpoint, and CRP levels of both the survivors and nonsurvivors following hip fracture surgery.

The methodological quality of the included studies was assessed using the Newcastle–Ottawa scale (NOS) [24]. The NOS comprises three domains, namely selection, comparability, and outcome, with a total maximum score of nine. High-quality studies had simultaneous scores of three or four in the selection domain, one or two in the comparability domain, and two or three in the outcome domain. Studies that scored zero or one in the selection and the outcome domain or zero in the comparability domain were regarded as being low quality. The two reviewers (Y.-C.L. and B.-K.C.) independently evaluated the methodological quality of the included studies, and any disagreements were resolved through team discussion with the third reviewer (Y.-P. C.).

### Outcomes

The primary outcomes of interest were the correlations of preoperative or postoperative CRP level with postoperative long-term mortality (≥ 6 months) and short-term mortality (≤ 30 days) in patients undergoing hip fracture surgery.

### Statistical analysis and data synthesis

The extracted data were meta-analyzed using Review Manager software (version 5.4; Cochrane Collaboration, Oxford, United Kingdom). Differences in preoperative and postoperative CRP levels between nonsurvivors and survivors were measured using mean differences (MDs) and 95% confidence intervals (CIs). For studies providing CRP data in the form of the median with full range or interquartile range, mathematical conversion to the mean with standard deviation was performed [25, 26]. A *P* value of < 0.05 indicated statistical significance. Heterogeneity among the included studies was examined using the Chi-squared test, Cochran’s Q test, and *I*^2^ test. Significance was set at *P* < 0.1 for the Cochran’s Q test and at *I*^2^ values of > 50% [27]. In the *z* test for equivalence, *P* < 0.05 indicated significance. Publication bias was evaluated through Egger’s statistical test, performed using the Comprehensive Meta-Analysis Software (Biostat, Englewood, NJ, USA).

## Results

**Fig. 1:**
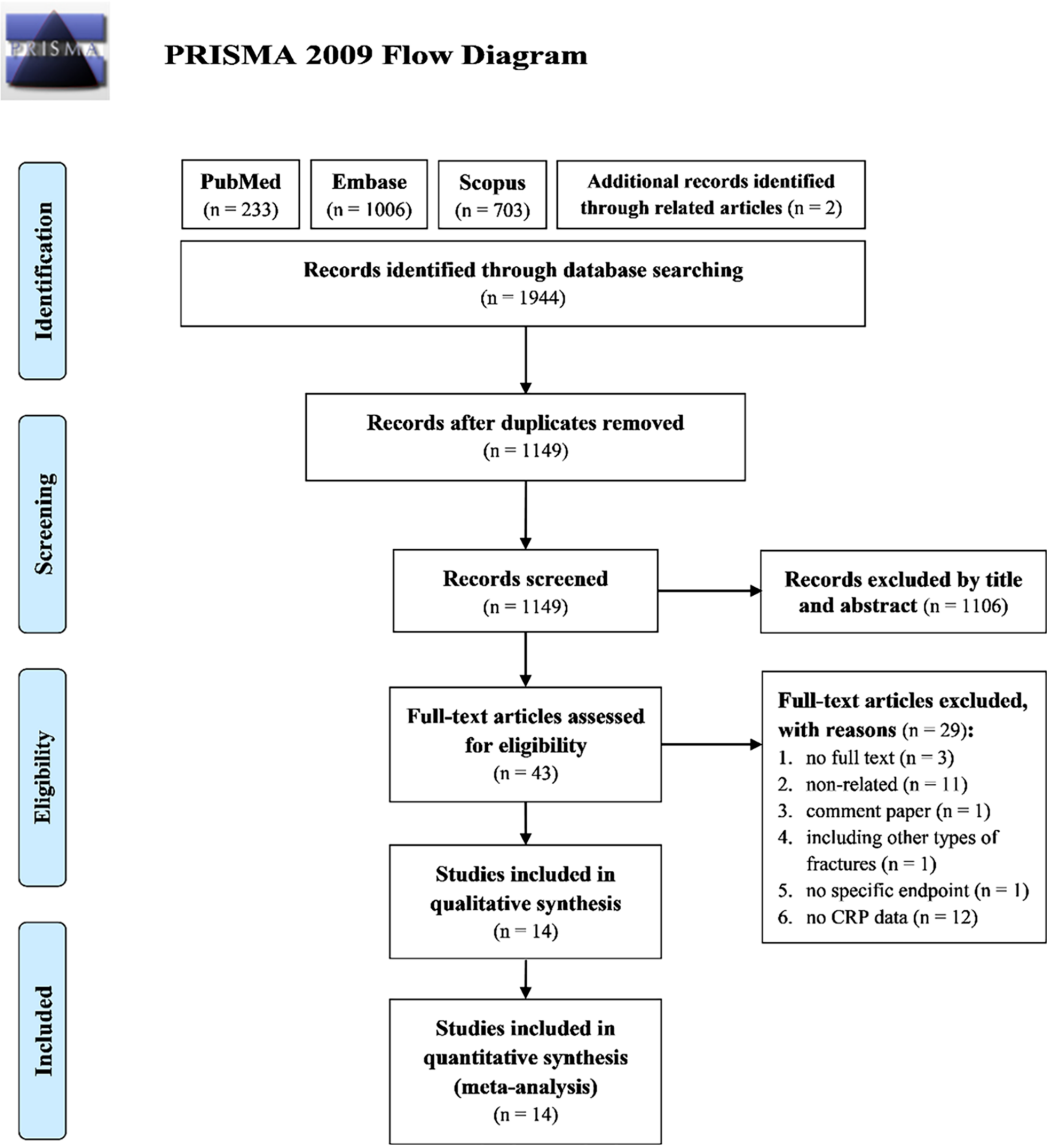
PRISMA flowchart of the selection of included studies

Figure 1 presents the flowchart of study screening and selection. The initial search of three databases (PubMed, Embase, and Scopus) yielded 1944 studies. After the removal of duplicates, 1149 studies were screened by examining their titles and abstracts, after which 1106 studies were excluded. For the remaining 43 studies, full-text evaluation was performed. Of these studies, 29 were excluded because full texts were not available for 3 studies, 11 were studies investigating topics than that of interest, 1 was a comment paper, 1 included patients with other types of fracture, 1 did not mention a specific endpoint, and 12 did not provide complete data on CRP levels. The remaining 14 studies were included in the meta-analysis [19–22, 28–37]

Table 1 presents the baseline characteristics of the included studies. The 14 studies had either a prospective or retrospective design, comprised 3986 patients in total, and were published between 2013 and 2022. Ten studies compared the preoperative or postoperative CRP levels of death and survival groups for a follow-up duration of ≥ 6 months [19, 20, 22, 28, 31, 32, 34–37], and four compared the preoperative CRP levels of the two groups for a follow-up duration of ≤ 30 days [21, 29, 30, 33].

**Table 1:** Baseline characteristics of the included studies

Author [Year]	Country	Design	Model	Sample size	Age (years) Mean ± SD/median (min–max)	Women (n, %)	Endpoint
*Long-term mortality* (≥ 6 months)							
Azevedo [2017]	Brazil	Prospective cohort	Preoperative CRP	D: 17	D: 82.9 ± 8.9	D: 7, 41.2%	6-month mortality
				S: 58	S: 78.4 ± 7.2	S: 42, 72.4%	
Capkin [2021]	Turkey	Retrospective cohort	Preoperative CRP	D: 58	78.74 ± 6.88	D: 25, 36.7%	1-year mortality
				S: 196		S: 124, 63.3%	
Choi [2021]	South Korea	Retrospective cohort	Postoperative CRP	D: 43	D: 82.5 ± 5.8	D: 27, 62.8%	1-year mortality
				S: 220	S: 80.0 ± 7.2	S: 163, 74.1%	
Colino [2018]	Spain	Prospective cohort	Preoperative CRP	D: 118	D: 88.1 ± 6.5	D: 86, 76.9%	1-year mortality
				S: 391	S: 84.8 ± 6.9	S: 317, 81.1%	
Gulin [2015]	Croatia	Prospective cohort	Preoperative CRP	D: 67	D: 86.0 (82.0–89.0)	D: 47, 70.1%	1-year mortality
				S: 169	S: 79.0 (73.0–85.3)	S: 130, 76.9%	
Gumieiro [2013]	Brazil	Prospective cohort	Preoperative CRP	D: 11	D: 82.5 ± 6.8	D: 8, 72.7%	6-month mortality
				S: 71	S: 80.0 ± 7.39	S: 54, 76.1%	
Kim [2016]	Korea	Retrospective cohort	Preoperative CRP	D: 109	D: 80.2 ± 8.0	D: 73, 67%	1-year mortality
			Postoperative CRP	S: 663	S: 79.3 ± 7.1	S: 507, 76.5%	
McLeod [2022]	United Kingdom	Retrospective cohort	Preoperative CRP	D: 94	D: 80.9 ± 9.6	D: 70, 74.5%	1-year mortality
				S: 235	S: 82.5 ± 10.0	S: 174, 74%	
Sedlář [2015]	Czechia	Prospective cohort	Preoperative CRP	D: 64	D: 82 ± 8	D: 47, 73.4%	5-year mortality
			Postoperative CRP	S: 40	S: 76 ± 8	S: 28, 70%	
Zhou [2021]	China	Retrospective cohort	Postoperative CRP	D: 26	N/A	D: 10, 38.5%	1-year mortality
				S: 98		S: 63, 64.3%	
*Short-term mortality *(≤ 30 days)							
Bae [2021]	Korea	Retrospective cohort	Preoperative CRP	D: 19	D: 82.5 ± 10.1	D: 9, 47.4%	Intrahospital mortality
				S: 443	S: 74.0 ± 16.3	S: 311, 70.2%	
Balta [2022]	Turkey	Retrospective cohort	Preoperative CRP	D: 30	83.09 ± 8.52	D: 19, 63.3%	30‑day mortality
				S: 135		S: 76, 56.3%	
Çiçek [2021]	Turkey	Retrospective cohort	Preoperative CRP	D: 30	D: 84.4 ± 6.2	D: 22, 73.3%	Intrahospital mortality
				S: 247	S: 81.7 ± 7.5	S: 181, 73.3%	
Niessen [2018]	Belgium	Retrospective cohort	Preoperative CRP	D: 16	D: 85.3 ± 9.1	N/A	Intrahospital mortality
				S: 318	S: 83.6 ± 7.4		

CRP, C-reactive protein; D, death group; max, maximum; min, minimum; N/A, not available; S, survival group; SD, standard deviation

The methodological quality of the included studies was assessed using the NOS, and the results are presented in the Additional file 1: Table S1. Of the 14 studies, 12 were determined to be high quality [19, 21, 22, 28–36] and 2 were regarded as low quality due to poor control for the baseline characteristics of the enrolled patients [20, 37].

### Correlation between preoperative CRP level and long-term mortality

**Fig. 2:**
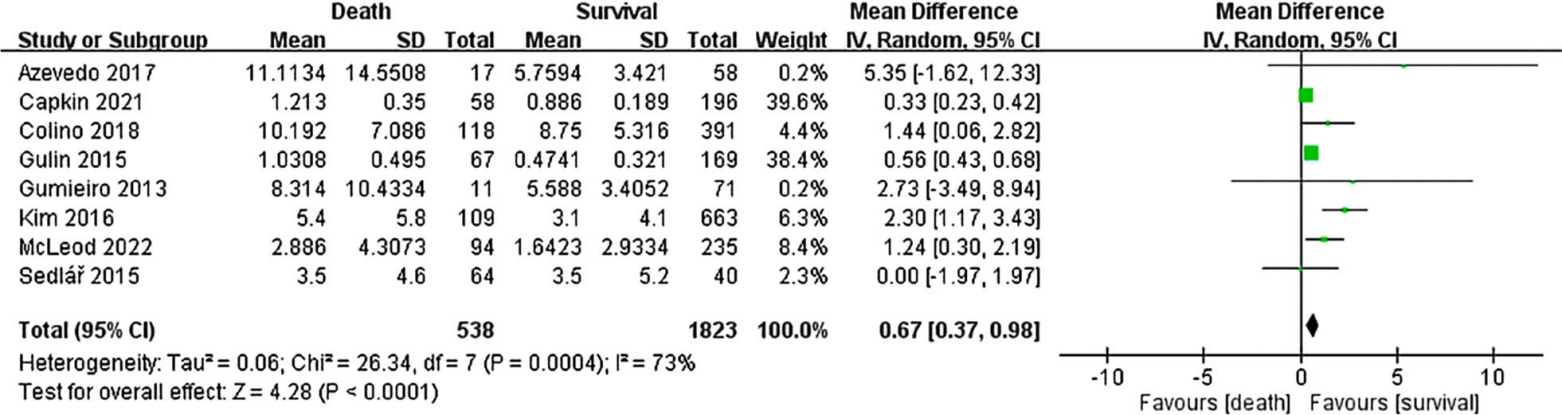
Forest plot of the mean difference in preoperative CRP level between the death and survival groups for a follow-up duration of ≥ 6 months

Eight of the included 14 studies, involving 2361 patients with hip fracture, compared preoperative CRP levels between the death and survival groups for a follow-up duration of ≥ 6 months (Fig. 2). Our findings revealed that preoperative CRP levels were significantly higher in the death group than in the survival group (MD: 0.67, 95% CI: 0.37–0.98; *P* < 0.0001). Significant heterogeneity was observed across the analyzed studies (*I*^2^: 73%, *P* < 0.05).

### Correlation between postoperative CRP level and long-term mortality

**Fig. 3:**
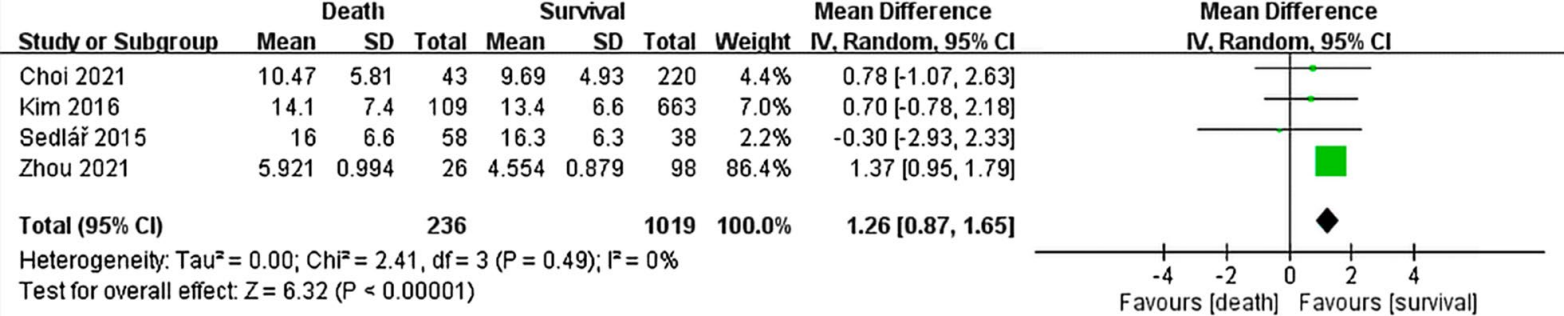
Forest plot of the mean difference in postoperative CRP level between the death and survival groups for a follow-up duration of ≥ 6 months

Four of the 14 studies, involving 1255 patients with hip fracture, compared postoperative CRP levels between the death and survival groups for a follow-up duration of ≥ 6 months (Fig. 3). Postoperative CRP levels were significantly higher in the death group than in the survival group (MD: 1.26, 95% CI: 0.87–1.65; *P* < 0.00001). Low heterogeneity was detected across the analyzed studies (*I*^2^: 0%, *P* = 0.49).

### Correlation between preoperative CRP level and short-term mortality

**Fig. 4:**
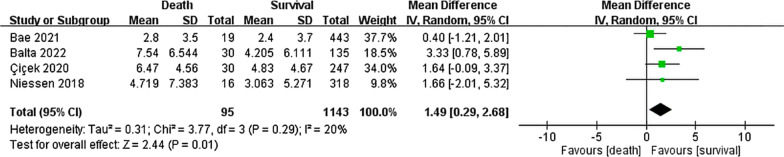
Forest plot of the mean difference in preoperative CRP level between the death and survival groups for a follow-up duration of ≤ 30 days

Four of the 14 studies, involving 1238 patients with hip fracture, compared preoperative CRP levels between the death and survival groups for a follow-up duration of ≤ 30 days (Fig. 4). Preoperative CRP levels were significantly higher in the death group than in the survival group (MD: 1.49, 95% CI: 0.29–2.68; *P* = 0.01). Low heterogeneity was observed across the analyzed studies (*I*^2^: 20%, *P* = 0.29).

### Publication bias

The funnel plot of the studies comparing preoperative CRP levels between the death and survival groups for a follow-up duration of ≥ 6 months is presented in the Additional file 1: Fig. S1. No publication bias was detected through Egger’s statistical test (*t* = 0.632, *P* = 0.55).

## Discussion

The present meta-analysis demonstrated that both preoperative and postoperative CRP levels were correlated with higher risk of long-term mortality in patients undergoing hip fracture surgery. Furthermore, preoperative CRP level was correlated with higher short-term mortality risk following hip fracture surgery.

Systemic inflammation is a prognostic predictor of hip fracture. The correlation of the level of CRP, the most widely used biomarker for detecting inflammation, with hip fracture mortality has been reported in numerous observational studies [18–20]. In our meta-analysis, those who died following hip fracture exhibited higher levels of CRP preoperatively and postoperatively, suggesting a higher degree of inflammation during the perioperative period in these patients. In addition to CRP, numerous proinflammatory cytokines, biochemical markers, and indices based on these biomarkers that are used to assess inflammation have also been reported to be correlated with mortality following hip fracture [13–15]. High-severity systemic inflammation may interfere with healing and recovery from the initial hip injury [38] and thus cause prolonged physiological stress; moreover, the presence of long-term, low-grade inflammation may also correlate with higher incidence of chronic diseases such as cardiovascular and neurodegenerative diseases [39, 40]. The aforementioned effect of inflammatory response on the human body may influence mortality following hip fracture.

Eight of the studies included in this meta-analysis reported significantly higher mean age in nonsurvivors who underwent hip fracture surgery in comparison with survivors (pooled mean age: 85.4 vs. 79.3 years) [20, 22, 28–32, 34], suggesting an effect of age on hip fracture mortality. Advanced age is a well-established prognostic factor of hip fracture. Age was also reported to be positively correlated with CRP level [41]. The mechanism underlying this phenomenon remains controversial, and the concept of “inflammaging” has been proposed as an explanation. In this concept, aging is characterized by chronic, low-grade inflammatory status; an elevated CRP level is associated with a prolonged inflammatory process caused by insufficient clearance of senescent cells and elevated transcriptional factor NF-κB [42]. In our study, following hip fracture, nonsurvivors were older and had a higher CRP level, suggesting that age may simultaneously correlate with higher baseline CRP level and higher mortality risk in patients with hip fracture.

In addition to advanced age, nutritional status is another prognostic predictor of hip fracture [43]. Hypoalbuminemia is one of the clinical indicators of both poor nutritional status and inflammation [44]. Of our included studies, albumin level was provided in 10, and 8 reported significantly lower albumin levels in nonsurvivors than in survivors following hip fracture surgery [19, 20, 29–34]. Several studies have also reported that lower albumin level was correlated with higher mortality risk in patients with hip fracture [43, 45]. Under inflammation, multiple proinflammatory cytokines, particularly interleukin-1, enhance the production of inflammatory reactants in the liver, including CRP; consequently, the albumin level may diminish because it is also produced by the liver [46]. This may explain why nonsurvivors of hip fracture in our meta-analysis simultaneously presented with a lower albumin level and higher CRP level. In brief, hypoalbuminemia may reflect a high degree of systemic inflammation and relatively poor nutritional status in patients with hip fracture and thus contribute to higher mortality risk in patients with this clinical presentation.

Studies have reported cardiovascular diseases to be one of the predominant causes of death for patients undergoing hip fracture surgery in both short- and long-term follow-ups [47, 48]. CRP is associated with cardiovascular diseases, constituting an independent risk factor and prognosis factor [49, 50]. Considering that inflammation is involved in the development of cardiovascular diseases, CRP may reflect the inflammatory reactions and the amount and activity of circulating proinflammatory cytokines associated with atherosclerotic processes [51]; that is, CRP is indirectly linked to cardiovascular diseases and is considered to be a bystander marker of vascular inflammation. Furthermore, evidence is accumulating that CRP has a direct role in the development or occurrence of cardiovascular diseases. CRP is capable of stimulating the release of proinflammatory factors and mediating the uptake of low-density lipoprotein into macrophages, which converts them into foam cells and leads to atherosclerosis [52]. Additionally, CRP may promote thrombosis, platelet deposition, and thrombus growth, contributing to acute cardiovascular events [53]. In summary, patients with hip fracture presenting with a higher baseline CRP level may be at higher risk of developing cardiovascular diseases, which leads to a higher mortality rate compared with that of patients with a lower baseline CRP level.

In our investigation of the correlation between preoperative CRP level and long-term hip fracture mortality, significant heterogeneity (*I*^2^: 73%, *P* < 0.1) was detected. The heterogeneity may be attributable to baseline variations in the enrolled patients across the analyzed studies, such as variations in age, comorbidities, fracture type, surgery type, blood sampling timing in the preoperative period, and time interval from admission to operation [54]. In addition, we performed mathematical conversion to obtain adequate CRP data for outcome pooling for some studies, which may also have affected the results and thereby the heterogeneity. Despite the high heterogeneity, this meta-analysis is currently the first and the most extensive study investigating the correlation between CRP level and hip fracture mortality. Our findings suggest the prognostic role of CRP in patients with hip fracture and its additional applications for orthopedic surgeons in clinical practice.

## Limitations

The limitations of this meta-analysis must be addressed. First, the number of included studies was small. Second, the retrospective design of numerous studies included in this meta-analysis may have introduced potential biases such as selection bias. Third, many confounding factors relating to CRP level and hip fracture mortality such as age, gender, and comorbidities were not adequately controlled for at baseline in some of the included studies, which may have adversely affected with the results of our meta-analysis. Fourth, because of insufficient data provided in the included studies, we could not perform analysis to obtain the pooled hazard ratio of higher CRP level and hip fracture mortality or to obtain a pooled CRP cutoff value with sensitivity and specificity for the prediction of hip fracture mortality; this may be a future research direction considering the easy accessibility and wide use of CRP. Further well-controlled observational studies are required to clarify the role of CRP as an independent prognostic predictor of hip fracture and to establish a cutoff value with acceptable sensitivity and specificity for clinical practice.

## Conclusions

This meta-analysis demonstrated the correlation between CRP level and postoperative mortality in patients undergoing hip fracture surgery. CRP may be a prognostic predictor for hip fracture in clinical practice. Further research is warranted to evaluate the ability of CRP to predict postoperative mortality in patients with hip fracture.

## Supplementary Information

**Additional file 1.** Additional file of Correlation between C-Reactive Protein and Postoperative Mortality in Patients Undergoing Hip Fracture Surgery: A Meta-Analysis;**Table S1 :** Methodological quality of the included studies; **Fig. S1:** Funnel plot of the studies comparing preoperative CRP levels between the death and survival groups for a follow-up duration of ≥6 months.

## Data Availability

All data generated or analyzed during this study are included in this published article and its Additional files.

## Abbreviations

CRP: C-reactive protein
NOS: Newcastle–Ottawa scale
MDs: Mean differences
CIs: Confidence intervals

## References

[1] Kannus P, Parkkari J, Sievänen H, Heinonen A, Vuori I, Järvinen M (1996). Epidemiology of hip fractures. Bone.

[2] Cooper C, Campion G, Melton LJ (1992). Hip fractures in the elderly: a world-wide projection. Osteoporos Int.

[3] Gullberg B, Johnell O, Kanis JA (1997). World-wide projections for hip fracture. Osteoporos Int.

[4] Veronese N, Maggi S (2018). Epidemiology and social costs of hip fracture. Injury.

[5] Maffulli N, Aicale R (2022). Proximal femoral fractures in the elderly: a few things to know, and some to forget. Medicina (Kaunas).

[6] Migliorini F, Maffulli N, Trivellas M, Eschweiler J, Hildebrand F, Betsch M (2022). Total hip arthroplasty compared to bipolar and unipolar hemiarthroplasty for displaced hip fractures in the elderly: a Bayesian network meta-analysis. Eur J Trauma Emerg Surg.

[7] Chen YP, Kuo YJ, Liu CH, Chien PC, Chang WC, Lin CY (2021). Prognostic factors for 1-year functional outcome, quality of life, care demands, and mortality after surgery in Taiwanese geriatric patients with a hip fracture: a prospective cohort study. Ther Adv Musculoskelet Dis..

[8] Abrahamsen B, van Staa T, Ariely R, Olson M, Cooper C (2009). Excess mortality following hip fracture: a systematic epidemiological review. Osteoporos Int.

[9] Chiang MH, Huang YY, Kuo YJ, Huang SW, Jang YC, Chu FL (2022). Prognostic factors for mortality, activity of daily living, and quality of life in taiwanese older patients within 1 year following hip fracture surgery. J Pers Med.

[10] Rose S, Maffulli N (1999). Hip fractures. An epidemiological review. Bull Hosp Jt Dis.

[11] Hu F, Jiang C, Shen J, Tang P, Wang Y (2012). Preoperative predictors for mortality following hip fracture surgery: a systematic review and meta-analysis. Injury.

[12] Xu BY, Yan S, Low LL, Vasanwala FF, Low SG (2019). Predictors of poor functional outcomes and mortality in patients with hip fracture: a systematic review. BMC Musculoskelet Disord.

[13] Chen YH, Chou CH, Su HH, Tsai YT, Chiang MH, Kuo YJ (2021). Correlation between neutrophil-to-lymphocyte ratio and postoperative mortality in elderly patients with hip fracture: a meta-analysis. J Orthop Surg Res.

[14] Sun T, Wang X, Liu Z, Chen X, Zhang J (2011). Plasma concentrations of pro- and anti-inflammatory cytokines and outcome prediction in elderly hip fracture patients. Injury.

[15] Wang ZC, Jiang W, Chen X, Yang L, Wang H, Liu YH (2021). Systemic immune-inflammation index independently predicts poor survival of older adults with hip fracture: a prospective cohort study. BMC Geriatr.

[16] Allin KH, Nordestgaard BG (2011). Elevated C-reactive protein in the diagnosis, prognosis, and cause of cancer. Crit Rev Clin Lab Sci.

[17] Liu F, Li L, Xu M, Wu J, Luo D, Zhu Y (2020). Prognostic value of interleukin-6, C-reactive protein, and procalcitonin in patients with COVID-19. J Clin Virol.

[18] Ekinci M, Bayram S, Gunen E, Col KA, Yildirim AM, Yilmaz M (2021). C-reactive protein level, admission to intensive care unit, and high american society of anesthesiologists score affect early and late postoperative mortality in geriatric patients with hip fracture. Hip Pelvis.

[19] Kim BG, Lee YK, Park HP, Sohn HM, Oh AY, Jeon YT (2016). C-reactive protein is an independent predictor for 1-year mortality in elderly patients undergoing hip fracture surgery: a retrospective analysis. Medicine (Baltimore).

[20] Menéndez-Colino R, Alarcon T, Gotor P, Queipo R, Ramírez-Martín R, Otero A (2018). Baseline and pre-operative 1-year mortality risk factors in a cohort of 509 hip fracture patients consecutively admitted to a co-managed orthogeriatric unit (FONDA Cohort). Injury.

[21] Niessen R, Bihin B, Gourdin M, Yombi JC, Cornu O, Forget P (2018). Prediction of postoperative mortality in elderly patient with hip fractures: a single-centre, retrospective cohort study. BMC Anesthesiol.

[22] Sedlář M, Kvasnička J, Krška Z, Tománková T, Linhart A (2015). Early and subacute inflammatory response and long-term survival after hip trauma and surgery. Arch Gerontol Geriatr.

[23] Moher D, Shamseer L, Clarke M, Ghersi D, Liberati A, Petticrew M (2015). Preferred reporting items for systematic review and meta-analysis protocols (PRISMA-P) 2015 statement. Syst Rev.

[24] Stang A (2010). Critical evaluation of the Newcastle-Ottawa scale for the assessment of the quality of nonrandomized studies in meta-analyses. Eur J Epidemiol.

[25] Luo D, Wan X, Liu J, Tong T (2018). Optimally estimating the sample mean from the sample size, median, mid-range, and/or mid-quartile range. Stat Methods Med Res.

[26] Wan X, Wang W, Liu J, Tong T (2014). Estimating the sample mean and standard deviation from the sample size, median, range and/or interquartile range. BMC Med Res Methodol.

[27] Thorlund K, Imberger G, Johnston BC (2012). Evolution of heterogeneity (I2) estimates and their 95% confidence intervals in large meta-analyses. PLoS ONE.

[28] Azevedo PS, Gumieiro DN, Polegato BF, Pereira GJ, Silva IA, Pio SM (2017). Goldman score, but not Detsky or Lee indices, predicts mortality 6 months after hip fracture. BMC Musculoskelet Disord.

[29] Bae SJ, Lee SH (2021). Computed tomographic measurements of the psoas muscle as a predictor of mortality in hip fracture patients: Muscle attenuation helps predict mortality in hip fracture patients. Injury.

[30] Balta O, Altınayak H, Gürler Balta M, Astan S, Uçar C, Kurnaz R (2022). Can C-reactive protein-based biomarkers be used as predictive of 30-day mortality in elderly hip fractures?A retrospective study. Ulus Travma Acil Cerrahi Derg.

[31] Capkin S, Guler S, Ozmanevra R (2021). C-reactive protein to albumin ratio may predict mortality for elderly population who undergo hemiarthroplasty due to hip fracture. J Invest Surg.

[32] Choi SU, Rho JH, Choi YJ, Jun SW, Shin YJ, Lee YS (2021). Postoperative hypoalbuminemia is an independent predictor of 1-year mortality after surgery for geriatric intertrochanteric femoral fracture: a retrospective cohort study. Medicine (Baltimore).

[33] Çiçek V, Cinar T, Hayiroglu MI, Kılıç Ş, Keser N, Uzun M (2021). Preoperative cardiac risk factors associated with in-hospital mortality in elderly patients without heart failure undergoing hip fracture surgery: a single-centre study. Postgrad Med J.

[34] Gulin T, Kruljac I, Kirigin L, Merc M, Pavić M, Trcin MT (2016). Advanced age, high β-CTX levels, and impaired renal function are independent risk factors for all-cause one-year mortality in hip fracture patients. Calcif Tissue Int.

[35] Gumieiro DN, Rafacho BP, Gonçalves AF, Santos PP, Azevedo PS, Zornoff LA (2013). Serum metalloproteinases 2 and 9 as predictors of gait status, pressure ulcer and mortality after hip fracture. PLoS ONE.

[36] McLeod G, Kennedy I, Simpson E, Joss J, Goldmann K (2022). Pilot project for a web-based dynamic nomogram to predict survival 1 year after hip fracture surgery: retrospective observational study. Interact J Med Res.

[37] Zhou J, Fu J, Zhao Q, Lin S, Zhu H (2021). Effect of neutrophil-to-lymphocyte ratio on short-term prognosis of elderly patients with hip fracture. Am J Transl Res.

[38] Bastian O, Pillay J, Alblas J, Leenen L, Koenderman L, Blokhuis T (2011). Systemic inflammation and fracture healing. J Leukoc Biol.

[39] Amor S, Puentes F, Baker D, van der Valk P (2010). Inflammation in neurodegenerative diseases. Immunology.

[40] Golia E, Limongelli G, Natale F, Fimiani F, Maddaloni V, Pariggiano I (2014). Inflammation and cardiovascular disease: from pathogenesis to therapeutic target. Curr Atheroscler Rep.

[41] Ferrucci L, Fabbri E (2018). Inflammageing: chronic inflammation in ageing, cardiovascular disease, and frailty. Nat Rev Cardiol.

[42] Chung HY, Kim DH, Lee EK, Chung KW, Chung S, Lee B (2019). Redefining chronic inflammation in aging and age-related diseases: proposal of the senoinflammation concept. Aging Dis.

[43] Malafarina V, Reginster JY, Cabrerizo S, Bruyère O, Kanis JA, Martinez JA (2018). Nutritional status and nutritional treatment are related to outcomes and mortality in older adults with hip fracture. Nutrients.

[44] Don BR, Kaysen G (2004). Serum albumin: relationship to inflammation and nutrition. Semin Dial.

[45] Li S, Zhang J, Zheng H, Wang X, Liu Z, Sun T (2019). Prognostic role of serum albumin, total lymphocyte count, and mini nutritional assessment on outcomes after geriatric hip fracture surgery: a meta-analysis and systematic review. J Arthroplasty.

[46] Sheinenzon A, Shehadeh M, Michelis R, Shaoul E, Ronen O (2021). Serum albumin levels and inflammation. Int J Biol Macromol.

[47] Chatterton BD, Moores TS, Ahmad S, Cattell A, Roberts PJ (2015). Cause of death and factors associated with early in-hospital mortality after hip fracture. Bone Joint J..

[48] von Friesendorff M, McGuigan FE, Wizert A, Rogmark C, Holmberg AH, Woolf AD (2016). Hip fracture, mortality risk, and cause of death over two decades. Osteoporos Int.

[49] Ridker PM, Buring JE, Rifai N, Cook NR (2007). Development and validation of improved algorithms for the assessment of global cardiovascular risk in women: the Reynolds Risk Score [published correction appears in JAMA. 2007 Apr 4;297(13):1433]. JAMA.

[50] Ridker PM, Cook N (2004). Clinical usefulness of very high and very low levels of C-reactive protein across the full range of Framingham Risk Scores. Circulation.

[51] Lagrand WK, Visser CA, Hermens WT, Niessen HW, Verheugt FW, Wolbink GJ (1999). C-reactive protein as a cardiovascular risk factor: more than an epiphenomenon?. Circulation.

[52] Li JJ, Fang CH (2004). C-reactive protein is not only an inflammatory marker but also a direct cause of cardiovascular diseases. Med Hypotheses.

[53] Badimon L, Peña E, Arderiu G, Padró T, Slevin M, Vilahur G (2018). C-reactive protein in atherothrombosis and angiogenesis. Front Immunol.

[54] Klestil T, Röder C, Stotter C, Winkler B, Nehrer S, Lutz M (2018). Impact of timing of surgery in elderly hip fracture patients: a systematic review and meta-analysis. Sci Rep.

