# Supplementary material for: Correlation between C-reactive protein and postoperative mortality in patients undergoing hip fracture surgery: a meta-analysis
Source: J Orthop Surg Res. 2023 Mar 9;18:182. doi: 10.1186/s13018-023-03516-y (PMC9996565; doi:10.1186/s13018-023-03516-y)
Supplement: Supplementary file 1 — Additional file 1. Additional file of Correlation between C-Reactive Protein and Postoperative Mortality in Patients Undergoing Hip Fracture Surgery: A Meta-Analysis;Table S1 : Methodological quality of the included studies; Fig. S1: Funnel plot of the studies comparing preoperative CRP levels between the death and survival groups for a follow-up duration of ≥6 months. [file 13018_2023_3516_MOESM1_ESM.docx]

*Additional file*

**Correlation between C-Reactive Protein and Postoperative Mortality in Patients Undergoing Hip Fracture Surgery: A Meta-Analysis**

Bing-Kuan Chen, Yu-Cheng Liu, Chun-Ching Chen, Yu-Ping Chen, Yi-Jie Kuo, Shu-Wei Huang

**Supplementary table****:** Methodological quality of the included studies

| Author [year] | Risk of bias assessment based on the **Newcastle–Ottawa Scale** | | | |
| --- | --- | --- | --- | --- |
|  | Selection | Comparability | Outcome | Total score |
| Azevedo et al. [2017] | *** | * | *** | 7 |
| Bae et al. [2021] | *** | * | *** | 7 |
| Balta et al. [2022] | *** | * | *** | 7 |
| Capkin et al. [2021] | **** | * | ** | 7 |
| Choi et al. [2021] | **** | * | *** | 8 |
| Çiçek et al. [2021] | **** | ** | *** | 9 |
| Colino et al. [2018] | *** | - | ** | 5 |
| Gulin et al. [2015] | *** | * | ** | 6 |
| Gumieiro et al. [2013] | *** | ** | *** | 8 |
| Kim et al. [2016] | *** | ** | *** | 8 |
| McLeod et al. [2022] | *** | * | *** | 7 |
| Niessen et al. [2018] | *** | * | *** | 7 |
| Sedlář et al. [2015] | *** | * | ** | 6 |
| Zhou et al. [2021] | **** | - | ** | 6 |


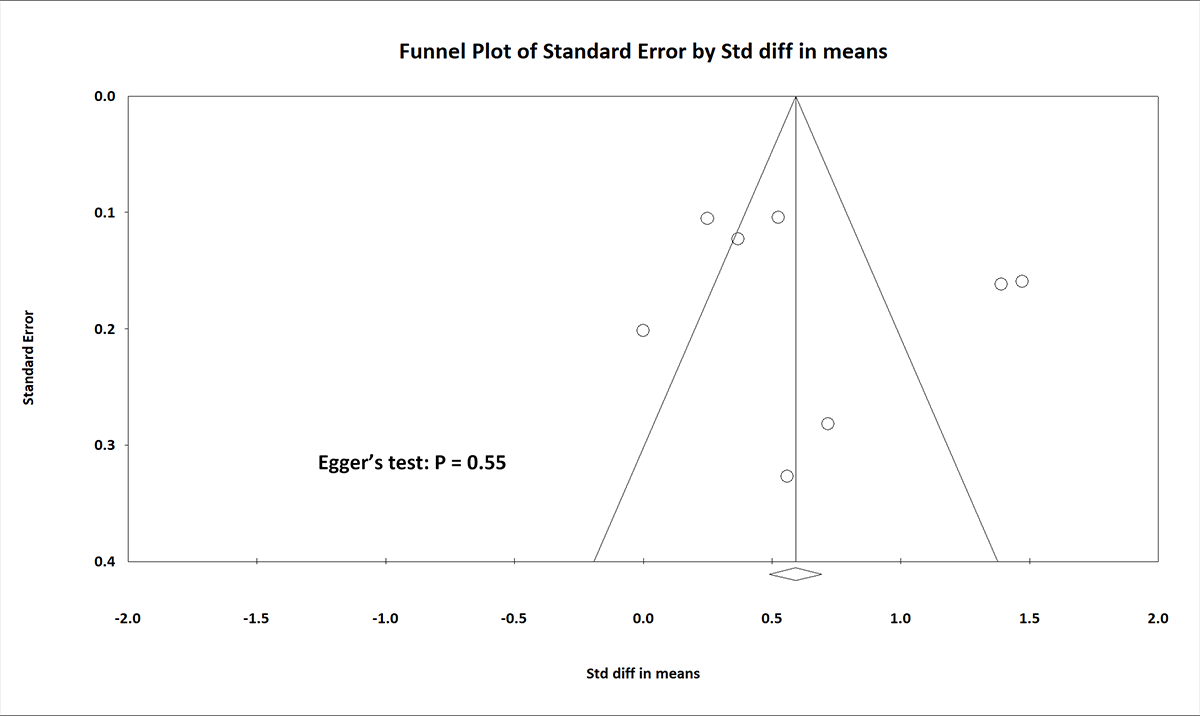


**Supplementary figure:** Funnel plot of the studies comparing preoperative CRP levels between the death and survival groups for a follow-up duration of ≥6 months
